# Preparation of an anti-NEK2 monoclonal antibody and its application in liver cancer

**DOI:** 10.1186/s12896-021-00717-3

**Published:** 2021-10-27

**Authors:** Qiuli Chen, Hui Li, Lichao Yang, Sha Wen, Xuejing Huang, Jiajuan Liu, Xiaoping Guo, Bing Hu, Gang Li, Min He

**Affiliations:** 1 School of Public Health, Guangxi Medical University, Nanning, 530021 China; 2 Laboratory Animal Center of Guangxi Medical University, Nanning, 530021 China; 3 Key Laboratory of High-Incidence-Tumor Prevention & Treatment (Guangxi Medical University), Ministry of Education, Nanning, 530021 China

**Keywords:** NEK2, Prokaryotic expression, Monoclonal antibody, Hepatocellular carcinoma, Cell cycle

## Abstract

**Background:**

Never in mitosis gene-A (NIMA)-related expressed kinase 2 (NEK2) is a serine/threonine protein kinase regulated by the cell cycle. The purpose of this study was to obtain NEK2 protein to prepare an anti-NEK2 monoclonal antibody (mAb) and explore the application of the anti-NEK2 mAb of therapeutic and diagnostic in hepatocellular carcinoma (HCC).

**Results:**

The *NEK2* gene sequence was cloned from the normal liver cell line HL7702, and the full-length *NEK2* gene sequence was cloned into the prokaryotic expression vector pET30a and transformed into *Escherichia coli* BL21 (DE3) cells. The recombinant fusion protein was obtained under optimized conditions and injected in BALB/c mice to prepare an anti-NEK2 mAb. By screening, we obtained a stable hybridoma cell line named 3A3 that could stably secrete anti-NEK2 mAb. Anti-NEK2 3A3 mAb was purified from ascites fluid. The isotype was IgG1, and the affinity constant (K_aff_) was 6.0 × 10^8^ L/mol. Western blot, indirect enzyme-linked immunosorbent assay (iELISA), immunofluorescence and immunocytochemical analyses showed that the mAb could specifically recognize the NEK2 protein. MTT assays showed that the mAb 3A3 could inhibit the proliferation of HCC cells. KEGG pathway analysis showed that NEK2 might affected pathways of the cell cycle. Moreover, NEK2-related genes were mainly enriched in the S and G2 phases and might act as tumor-promoting genes by regulating the S/G2 phase transition of HCC cells.

**Conclusions:**

An anti-NEK2 mAb with high potency, high affinity and high specificity was prepared by prokaryotic expression system in this study and may be used in the establishment of ELISA detection kits and targeted treatment of liver cancer.

**Supplementary Information:**

The online version contains supplementary material available at 10.1186/s12896-021-00717-3.

## Background

Never in mitosis Gene-A (NIMA)-related expressed kinase 2 (NEK2), a serine/threonine protein kinase regulated by the cell cycle, is localized in centrosomes [1]. NEK2 regulates the cell cycle and mitosis through centrosome separation during mitosis. Its expression level reaches a peak in the S and G2 phases of the cell cycle [2]. Abnormal expression of NEK2 can lead to chromosomal division abnormalities, resulting in chromosome instability and the formation of polyploid cells, thus promoting tumorigenesis [3].

In 2002, Wai [4] used oligonucleotide chips to analyze solid tumor cell lines in children and found that NEK2 was overexpressed in Ewing's sarcoma in children, suggesting for the first time that abnormal expression of NEK2 was correlated with the occurrence and development of tumors. Subsequently, many studies reported that NEK2 was highly expressed in a variety of malignant tumors, such as liver cancer [5], gastric cancer [6], head and neck squamous cell carcinoma [7], and bladder cancer [8] and was related to the occurrence and malignant transformation of tumors. Our previous study found that NEK2 was highly expressed in liver cancer cells and tissues, and the overexpression of NEK2 was related to clinicopathological features and poor prognosis of patients, suggesting that NEK2 may be an important biomarker of HCC [9]. Preparing an anti-NEK2 monoclonal antibody (mAb) and using an mAb to establish an enzyme-linked immunosorbent assay (ELISA) kit can be applied in the detection of liver cancer in the population and improve the detection rate of liver cancer, especially in high-risk populations. In this sense, it is important to develop sensitive monoclonal antibodies against NEK2 for the diagnosis of liver cancer.

NEK2 overexpression activated Akt, an oncogene associated with most malignancies, so NEK2 could be a novel target for suppressing tumor-related pathways [1, 2]. Another study found that in mice with subcutaneous transplantation of pancreatic cancer cells, after injection of NEK2 siRNA around the tumor, tumor growth was significantly inhibited, the survival time of mice with peritoneal metastasis was prolonged, and the survival rate was increased [10]. Kokuryo [11] used siRNA to interfere with the expression of NEK2 in biliary epithelial cancer cells and fibroblasts, and the results showed that the inhibition of NEK2 could significantly inhibit the proliferation of biliary epithelial cancer cells but had no effect on the proliferation of normal fibroblasts, suggesting that the targeted inhibition of NEK2 is highly selective for tumor cells. Therefore, NEK2 may be a new target for cancer treatment.

Currently, common cancer treatments are not satisfactory, mainly because of the weak targeting of treatment, lack of good selectivity, and strong side effects. Moreover, some treatments are too expensive to be suitable for most patients. The emergence and development of monoclonal antibodies provides a good solution to this problem. In recent years, monoclonal antibodies have been developed into a powerful tool for disease treatment, drug development and other applications [12]. Studies have shown that monoclonal antibodies targeting HER2 can be widely used in the treatment of breast cancer [13]. Therefore, the development and application of sensitive monoclonal antibodies against NEK2 for targeted tumor therapy may be a potentially effective approach for cancer prevention and treatment. However, there have been no reports on the preparation of monoclonal antibodies against NEK2.

In this study, a prokaryotic expression system was used to prepare anti-NEK2 mAbs with high potency, high specificity and high affinity. An anti-NEK2 mAb was applied for immunofluorescence and immunocytochemical analyses, providing an experimental basis for the development of a NEK2 rapid diagnostic kit and immunocolloidal gold detection strip. Moreover, the anti-NEK2 mAb was co-cultured with hepatocellular carcinoma (HCC) cells to investigate its effect on the proliferation of HCC cells and to provide a basis for NEK2-targeted drug research.

## Methods

### Materials and chemicals

The expression plasmid pET30a was provided by Guangxi University (Guangxi, China) and stored in our laboratory. The HCC cell lines HepG2 and Hep3B, the normal liver cell line HL7702, and the myeloma cell line SP2/0 were obtained from The Cell Resource Center of Shanghai Institute of Biotechnology, Chinese Academy of Sciences (Shanghai, China) and stored in our laboratory. *Escherichia coli* BL21 (DE3) and trans5α competent cells were obtained from TransGen Biotechnology Co., Ltd. (Beijing, China). RNA extraction kits and reverse transcription kits were purchased from TaKaRa (Japan). PCR primers were synthesized by Beijing Ruibo Xingke Biotechnology Co., Ltd. (Beijing, China). Plasmid DNA extraction kits and DNA purification kits were purchased from OMEGA (USA). A BCA protein concentration assay kit was purchased from Biyun Tian Company (Shanghai, China). Pierce NHS-activated agarose dry resin, HRP-labeled goat anti-mouse IgG and FITC-labeled goat anti-mouse IgG were purchased from Thermo Fisher Scientific (Waltham, MA, USA). Hypoxanthine, aminopterin and thymidine supplement (HAT), hypoxanthine and thymidine supplement (HT), mouse monoclonal antibody typing kit, and polyethylene glycol solution (PEG) were purchased from Sigma (St. Louis, Missouri, USA). The SP kit was purchased from Origene (Beijing, China). BALB/c mice were obtained from Shanghai Slac Laboratory Animal Co., Ltd. (Shanghai, China). All mice were housed in specific pathogen-free facilities and cared for in Laboratory Animal Center of Guangxi Medical University. The mice were euthanized by spine dislocation. All animal experiments obeyed the protocols approved by the Animal Ethics Committee of the Guangxi Medical University. And the study was carried out in accordance with ARRIVE guidelines. In addition, all methods were carried out in accordance with relevant guidelines and regulations.

### Preparation and identification of recombinant NEK2 fusion protein

Full length gene sequence of human *NEK2* was retrieved from NCBI database (Gene ID: 4751). The *NEK2* gene sequence was cloned from the normal liver cell line HL7702, and the *NEK2* target gene was amplified by PCR, digested by BamHI/Sa1I, and then inserted into the plasmid pET30a to transform *E. coli* BL21 (DE3) cells. Moreover, the positive clone was induced by isopropyl-β-D-thiogalactopyranoside (IPTG), and the target proteins were expressed. For improved expression of NEK2 protein, the induction conditions of temperature (18 °C, 28 °C, 37 °C, 42 °C), IPTG concentration (0.2 mmol/L, 0.4 mmol/L, 0.6 mmol/L, 0.8 mmol/L, 1.0 mmol/L) and time (4 h, 8 h, 12 h, 16 h, 20 h, 24 h, 28 h, 32 h, 36 h) were optimized. According to the above optimized conditions, the expressed recombinant human NEK2 protein was subjected to 12% SDS-PAGE, stained with 250 mmol/L KCl and the cut gels were placed in PBS buffer and shaken overnight at 4 °C. The next day, the upper layer of liquid was the purified human NEK2 protein solution and subjected to 12% SDS-PAGE and analyzed by Coomassie brilliant blue staining. Finally, the concentrations of NEK2 protein were detected by a BCA protein concentration assay kit. The protein purity was analyzed by a gel imaging system, and the purified human NEK2 protein was identified by AB Sciex 4 800 Plus mass spectrometry and Western blot using an anti-His monoclonal antibody as the primary antibody.

### Preparation of anti-NEK2 mAb

Three five-week-old SPF female BALB/c mice which randomly selected were subcutaneously injected at multiple sites. The mice were marked in order before the experiment and injected in the same order each time. The first injection contained 150 µg NEK2 fusion protein mixed with an equal volume of complete Freund’s adjuvant. Immunizations were given at intervals of two weeks. The second, third and fourth immunizations contained 80 µg NEK2 fusion protein mixed with an equal volume of incomplete Freund's adjuvant. To decrease potential confounders, the animals/cages were returned to their original positions at the end of each experiment. After immunization, there were no significant differences in mental status, behavioral characteristics and growth status of mice compared to non-immunized mice. One week after the fourth injection, blood was extracted from the tail of the mice and tested by indirect enzyme-linked immunosorbent assays (iELISAs). NEK2 protein (1.5 µg/mL) was coated in a 96-well plate. The mouse antiserum was diluted with a threefold dilution ratio (1:000 ~ 1:243,000), and a negative control was established. After incubation with HRP-labeled goat anti-mouse IgG (1:10,000), TMB was added to each well for incubation. Finally, the reaction was stopped by 2 M H_2_SO_4_, and the absorbance value at 450 nm was determined. The serum titer of immunized mice was taken as the highest dilution ratio of positive serum (P)/negative serum (N) OD450 nm > 2.1 [14].

The mouse that had a higher titer was injected intraperitoneally with 100 μg of the purified recombinant NEK2 protein. Three days later, the splenocytes of the immunized BALB/c mice were mixed with SP2/0 myeloma cells using PEG. Positive hybridoma cells were screened with HAT medium by iELISAs. The positive hybridoma cells were screened at least three times by the subclone method until hybridoma cells that could stably secrete the anti-NEK2 mAb were obtained. Furthermore, isotypes were identified with the mouse monoclonal antibody typing kit.

Eight-week-old SPF female BALB/c mice were intraperitoneally injected with 0.5 mL of sterile liquid paraffin oil, and 7 days later, approximately 1 × 10^6^ hybridoma cells were injected into the mouse abdominal cavity. Ascites fluid was extracted when the abdomen of the mice was enlarged, and the supernatant was collected. The mAb was purified by Pierce NHS-activated agarose dry resin, the titer was detected by iELISAs, and isotypes were identified with a mouse monoclonal antibody typing kit.

### Affinity constant (K_aff_) determination of anti-NEK2 mAb

NEK2 proteins at 0.25 µg/mL, 0.5 µg/mL, 1 µg/mL, and 2 µg/mL were coated in a 96-well plate. Anti-NEK2 mAb as the primary antibody was serially diluted at a double dilution ratio (1:000 ~ 1:1,024,000), while the negative control was incubated with 1% BSA. The subsequent procedures were consistent with the protocol of the iELISA above. The calculation method of K_aff_ was the same as that reported in a previous study [15].

### Western blot and iELISA

Purified His-NEK2 proteins (20 ng, 50 ng and 100 ng) were used as samples, and 20 ng human Serum Amyloid A4 (SAA4) protein, which was generated and purified with the same methods as NEK2 protein in this study, and BSA protein were selected as controls. Anti-NEK2 mAb (1:10 000) as the primary antibody was incubated, and HRP-labeled goat anti-mouse IgG (1:10,000) as the secondary antibody was incubated. Finally, chemiluminescence was used for detection.

His-NEK2, His-SAA4 and BSA proteins (1.5 μg/mL) were coated in a 96-well plate, and PBS was used as a negative control. Anti-NEK2 mAb (1:10,000) as the primary antibody was incubated. The subsequent procedures were consistent with the ELISA protocol described above.

### Immunofluorescence assay and immunocytochemical staining assay

HL7702, HepG2 and Hep3B cells were fixed with 4% paraformaldehyde. After the samples were blocked with 1% BSA, an anti-NEK2 mAb (1:500) was added for incubation. After incubation with FITC-labeled goat anti-mouse IgG (1:1000) as the secondary antibody, NEK2 subcellular localization was observed by a Zeiss LSM 800 laser confocal scanning microscope.

After HL7702, HepG2 and Hep3B cells were fixed with 4% polyformaldehyde, immunocytochemical staining was performed using a SP kit. Anti-NEK2 mAb (1:1000) as the primary antibody was added. In addition, EVOS software was used for observation. The expression of NEK2 mRNA in liver cancer cells was detected by real-time PCR, and total RNA of HL7702, HepG2 and Hep3B cells was extracted by TRIzol. Real-time PCR was performed according to the instructions of the reverse transcription kit. The results were analyzed by the 2^−△△CT^ method [9]. Finally, immunocytochemical staining results were compared with real-time PCR results. Moreover, we analyzed the expression levels of NEK2 between normal liver tissue and primary liver tumor tissue by the UALCAN database. UALCAN (http://ualcan.path.uab.edu/index.html) is a web-based tool that allows researchers to perform analyses of gene expression levels from The Cancer Genome Atlas (TCGA).

### MTT assay

Approximately 3 × 10^3^ HepG2 and Hep3B cells were inoculated in a 96-well plate. Anti-NEK2 mAb at final concentrations of 5 µg/mL, 25 µg/mL, 50 µg/mL and 100 µg/mL was added as the experimental group, and the culture medium was added as the control group. After 0 h, 24 h, 48 h and 72 h, 5 mg/mL MTT was added to each well for 4 h. DMSO was added to dissolve the crystal violet. Finally, the absorbance value at 490 nm was determined. Cell inhibition rate (%) = (OD value of control group -OD value of experimental group)/OD value of control group × 100%.

### Co-expression and pathways analysis

Co-expressed genes of NEK2 were analyzed from TCGA using cBioPortal (http://www.cbioportal) [16], and co-expressed genes of NEK2 were evaluated by Spearman's correlation > 0.3 and p-value < 0.05. KEGG pathways related to the top 500 co-expressed genes of NEK2 were analyzed by DAVID (https://david.ncifcrf.gov/) online software. The resulting pathway map was drawn using KEGG [17].

### Statistics

SPSS 17.0 software was used for statistical analysis. Measurement data are expressed as the mean ± standard deviation $(\overline{x} \pm s)$. Independent sample T-tests were used to compare the mean between two independent samples. Differences were considered statistically significant if *P* < 0.05.

## Results

### Construction and identification of the recombinant plasmid pET30a-*NEK2*

**Fig. 1:**
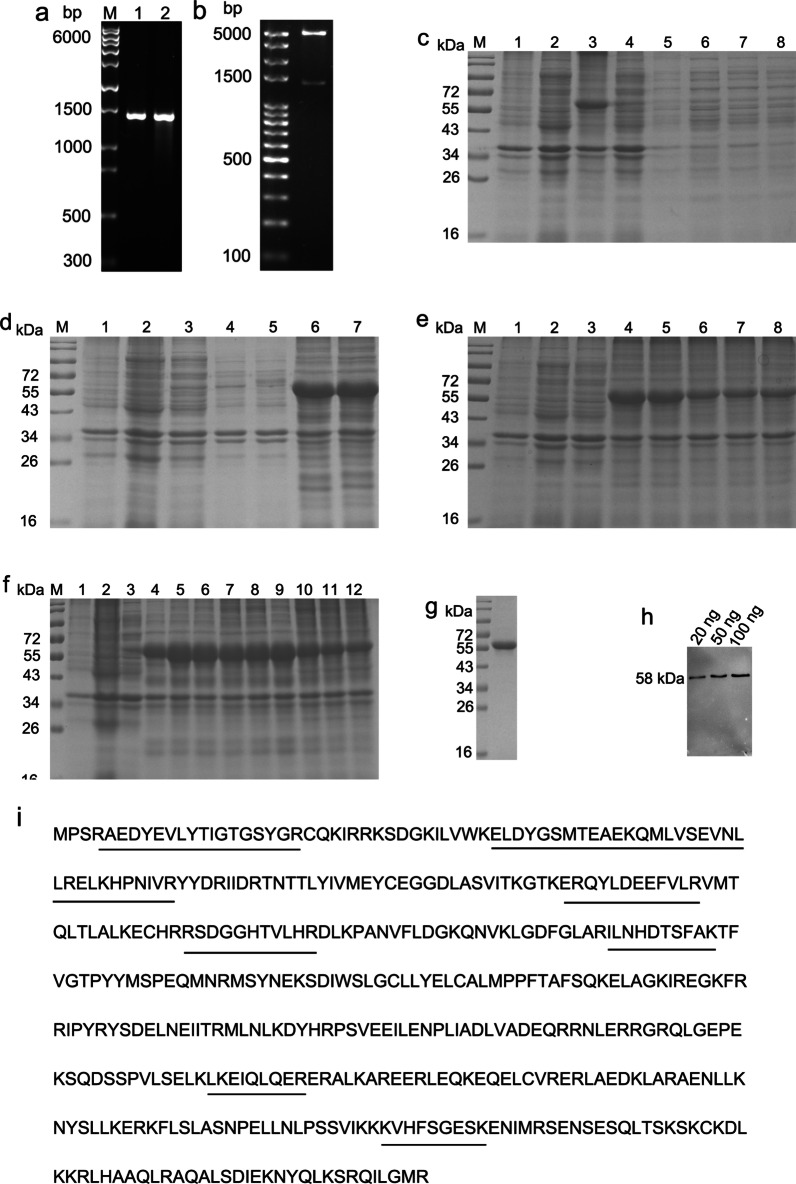
Cloning and Expression of NEK2 in prokaryotic system. **a** The construction of the pET30a-*NEK2* vector. M: DNA marker, 1: PCR amplification product of the *NEK2* gene, 2: Colony PCR detection. **b** BamHI and Sa1I enzymatic digestion of the recombinant plasmid. **c** Expression of the recombinant human NEK2 protein. M: marker, 1: Precipitate of the pET30a group with induction, 2: Precipitate of the pET30a group without induction, 3: Precipitate of the pET30a-*NEK2* group with induction, 4: Precipitate of the pET30a-*NEK2* group without induction, 5: Supernatant of the pET30a group with induction, 6: Supernatant of the pET30a group without induction, 7: Supernatant of the pET30a-*NEK2* group with induction, 8: Supernatant of the pET30a-*NEK2* group without induction. **d** Expression of recombinant human NEK2 protein at different temperatures. M: marker, 1: pET30a was induced, 2: pET30a was not induced, 3: pET30a-*NEK2* was not induced, 4–7: pET30a -*NEK2* was induced at 18 °C, 28 °C, 37 °C, and 42 °C. **e** Expression of recombinant human NEK2 protein at different IPTG concentrations. M-3: The same as the (**d**), 4–8: pET30a-*NEK2* was induced with 0.2 mmol/L, 0.4 mmol/L, 0.6 mmol/L, 0.8 mmol/L, and 1.0 mmol/L IPTG. **f** Expression of recombinant human NEK2 protein at different times. M-3: The same as the (**d**), 4–12: pET30a -*NEK2* was induced at 4 h, 8 h, 12 h, 16 h, 20 h, 24 h, 28 h, 32 h, and 36 h. **g** SDS-PAGE analysis of recombinant human NEK2 protein purified. **h** Western blot analysis of purified NEK2 protein using an anti-His monoclonal antibody. **i** Mass spectrometry identification of the recombinant human NEK2 protein with Mascot alignment. For improved clarity and conciseness, cropped areas of the blot are shown. The full length (uncut) blot image is shown in Additional file 2: Fig. S1–S8, respectively

The results of 1.5% agarose gel electrophoresis showed that the PCR amplification products had a specific band at approximately 1338 bp, similar to the size of the target gene *NEK2* (Fig. 1a, lane 1). The pET30a-*NEK2* recombinant plasmid was transformed into *E. coli* trans5ɑ cells, and PCR confirmed that the positive clones were consistent with the size of the *NEK2* gene (Fig. 1a, lane 2). With BamHI and SalI double enzyme digestion, an approximately 5500 bp vector band and 1338 bp target gene band were observed, consistent with the expected gene fragment size (Fig. 1b). The positive clonal bacterial solution was sent for sequencing, and the results were consistent with the sequence of the *NEK2* target gene (Additional file 1: Data S1), indicating that the recombinant expression vector of pET30a-*NEK2* was successfully constructed.

### Expression and identification of the NEK2 fusion protein

The recombinant strain was induced by IPTG, and the results showed that the precipitated components of the recombinant strain after IPTG induction exhibited specific clear bands at approximately 58 kDa (Fig. 1c), indicating that the induced recombinant human NEK2 protein mainly existed in the form of inclusion bodies. Through the optimization of protein expression conditions, we found that the best yield of the fusion protein could be obtained when the recombinant expression vector was induced by 0.2 mmol/L IPTG at 37 °C for 8 h (Fig. 1d–1f).

The NEK2 protein was purified with 250 mmol/L KCl staining, showing a clear band at approximately 58 kDa with no obvious heterotopic bands around it (Fig. 1g), which was consistent with the size of the target NEK2 protein band. The purity of the protein analyzed by the gel imaging system was greater than 95%. The purified protein was detected by Western blots, and the protein could be specifically recognized by an anti-His monoclonal antibody (Fig. 1h). The recombinant human NEK2 protein was analyzed by mass spectrometry. The results were compared with Mascot (Fig. 1i), showing that eight polypeptides (underlined part) were identified by mass spectrometry that matched the amino acid sequence of the human NEK2 protein. The concentration of the purified NEK2 protein was 2.21 mg/mL, as shown by a BCA kit.

### Preparation of anti-NEK2 mAb

**Fig. 2:**
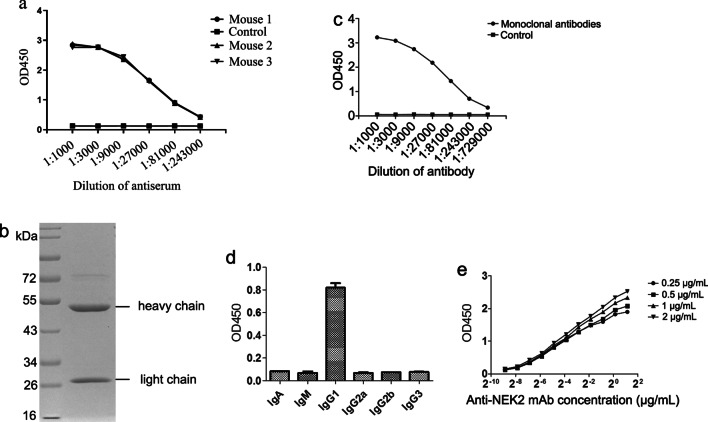
Preparation of anti-NEK2 mAb. **a** Serum antibody titer of mouse after immunization with NEK2 protein was tested by iELISA. **b** SDS-PAGE analysis of 3A3 mAb purified. For improved clarity and conciseness, cropped areas of the gel are shown. The full length (uncut) gel image is shown in Additional file 2: Fig. S9. **c** The titer of purified anti-NEK2 3A3 mAb was tested by iELISA. **d** The isotype of purified anti-NEK2 3A3 mAb was tested by iELISA. **e** The affinity of purified anti-NEK2 3A3 mAb was tested by iELISA

After immunizing BALB/c mice with NEK2 protein four times, we detected the serum titer of the mice. The results showed that the antiserum titer of the mice was above 1:243,000 (Fig. 2a), indicating that the immune effect of the mouse was good, and the spleen cells of the mouse could be used for cell fusion with S/P20 myeloma cells. We did two fusion experiments. The fusion efficiency index was 4.2% in the first experiment in which no hybridoma cell line was screened out. In the second experiment, the fusion efficiency index was 29.6%, and six hybridoma cell lines stably secreting anti-NEK2 antibody were screened out, which are named 2C9, 2D8, 3A3, 3B3, 7G4 and 10D6, respectively. The cell line of 3A3 showed a good titer, therefore 3A3 was chosen for the further experiments.

After purification of the anti-NEK2 3A3 mAb, there were two distinct bands at 55 kDa and 25 kDa (Fig. 2b), which were the heavy and light chains of antibody molecules, respectively. The purity of the antibody was greater than 90%, the titer was above 1:729,000 (Fig. 2c), and the isotype was IgG1 (Fig. 2d). iELISA was used to detect the binding activity of anti-NEK2 3A3 mAb and the human NEK2 protein. The results showed that the EC50 values corresponding to 0.25 µg/mL, 0.5 µg/mL, 1 µg/mL and 2 µg/mL protein concentrations were 0.0533 µg/mL, 0.0986 µg/mL, 0.106 µg/mL and 0.116 µg/mL, respectively. According to the formula calculation, K_aff_ was 6.0 × 10^8^ L/mol (Fig. 2e).

### Specific detection and application of anti-NEK2 3A3 mAb

**Fig. 3:**
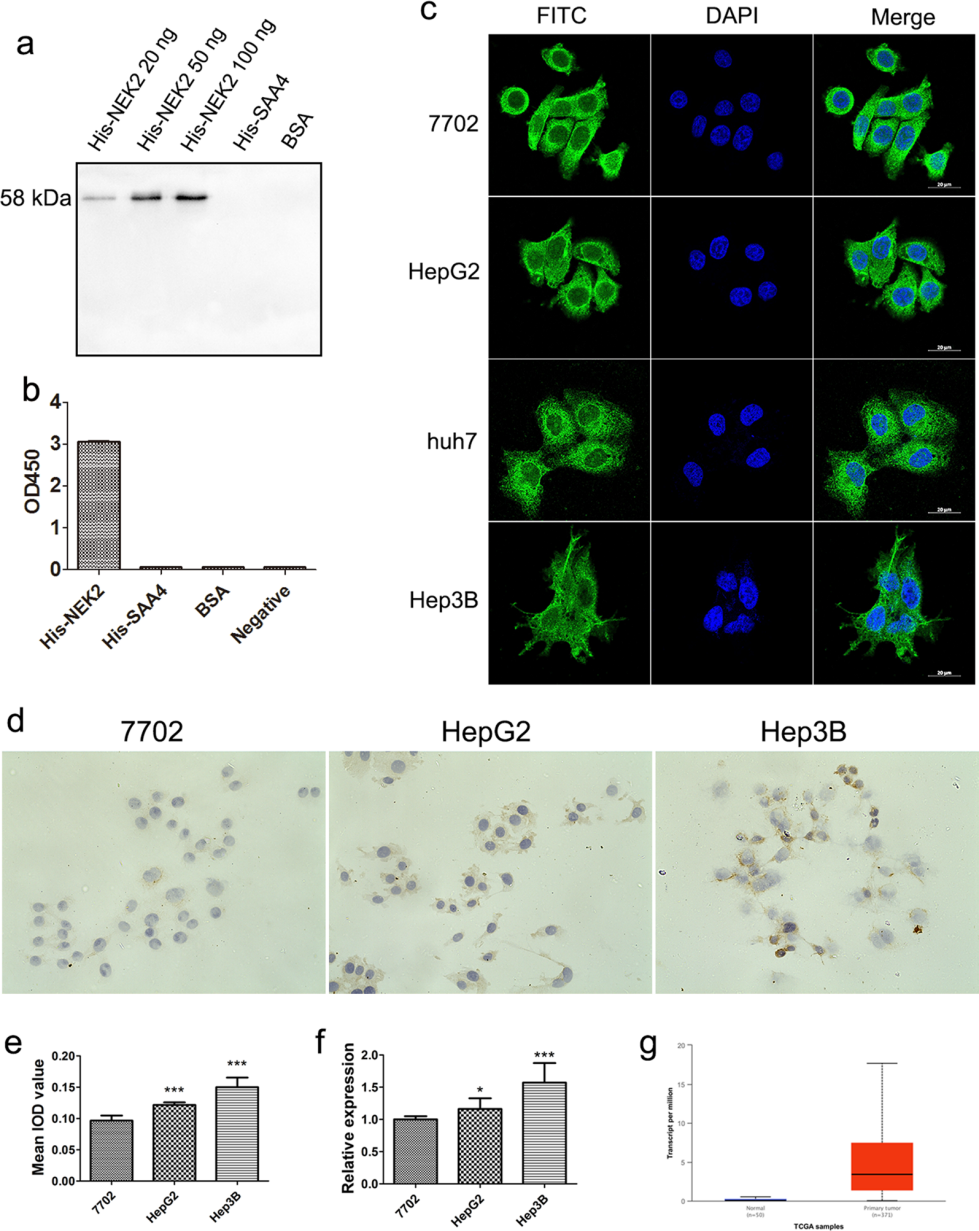
Specificity detection of anti-NEK2 mAb. **a** Analysis of the antigenic specificity of anti-NEK2 mAb by Western blot. For improved clarity and conciseness, cropped areas of the blot are shown. The full length (uncut) blot image is shown in Additional file 2: Fig. S10. **b** Analysis of the antigenic specificity of the anti-NEK2 mAb by ELISA. **c** Detection of NEK2 subcellular localization by immunofluorescence assay at HCC cell lines HepG2, Huh7 and Hep3B, and the normal liver cell line HL7702. Cell nuclei were stained with DAPI (blue). **d** Detection of NEK2 expression in HCC cells by immunocytochemical staining at HCC cell lines HepG2, Hep3B and the normal liver cell line HL7702. **e** Quantitative analysis of immunocytochemical staining. **f** *NEK2* mRNA relative expression at HCC cell lines HepG2, Hep3B and the normal liver cell line HL7702. **g** The expression levels of NEK2 between normal liver tissues and primary liver tumor tissues by TCGA database were analyzed

Western blot, iELISA, immunofluorescence and immunocytochemical staining were used to detect the specificity of the anti-NEK2 3A3 mAb. The Western blot results showed that the purified His-NEK2 protein had specific bands at 58 kDa, and the band brightness increased with increasing NEK2 content, while His-SAA4 and BSA proteins had no bands (Fig. 3a). The iELISA results showed that the anti-NEK2 3A3 mAb could specifically recognize the NEK2 protein, which was consistent with the Western blot results (Fig. 3b). Immunofluorescence assays were used to detect the subcellular localization in HL7702, HepG2 and Hep3B cells. The results showed that green fluorescence was mainly distributed in the cytoplasm with a small amount in the nucleus, indicating that the NEK2 protein was mainly located in the cytoplasm (Fig. 3c). Immunocytochemical staining was used to detect the expression of the NEK2 protein in HCC cells. We found that the positive expression of the NEK2 protein in HepG2 and Hep3B HCC cells was significantly higher than that in the normal liver cell line HL7702, and its immune reactants were mainly located in the cytoplasm (Fig. 3d). Image-Pro Plus 6.0 software was used for quantitative analysis of the immunocytochemical staining images, and the results showed that the NEK2 protein expression levels in HepG2 (0.1217 ± 0.00408; 95%Cl: -0.03330, -0.01670; *P* = 0.000) and Hep3B (0.15 ± 0.1549; 95%Cl: -0.06998, -0.03669; *P* = 0.000) HCC cells were significantly higher than those in normal liver HL7702 cells (0.0967 ± 0.00816) (Fig. 3e). Consistent with the real-time PCR results, the NEK2 mRNA expression levels in HepG2 (1.1611 ± 0.16602; 95%Cl: -0.28965, -0.03035; *P* = 0.021) and Hep3B (1.5700 ± 0.30381; 95%Cl: -0.80339, -0.33439; *P* = 0.000) HCC cells were significantly higher than those in the normal liver HL7702 cells (1.0011 ± 0.04833) (*P* < 0.05) (Fig. 3f). In addition, a higher expression level of NEK2 was found in primary liver tumor tissues than in normal liver tissues (Fig. 3g). Western blot, iELISA, immunofluorescence and immunocytochemical staining results all showed that the anti-NEK2 3A3 mAb had good antigen specificity.

### MTT assays were used to detect the effect of anti-NEK2 3A3 mAb on the proliferation of HCC cells

**Fig. 4:**
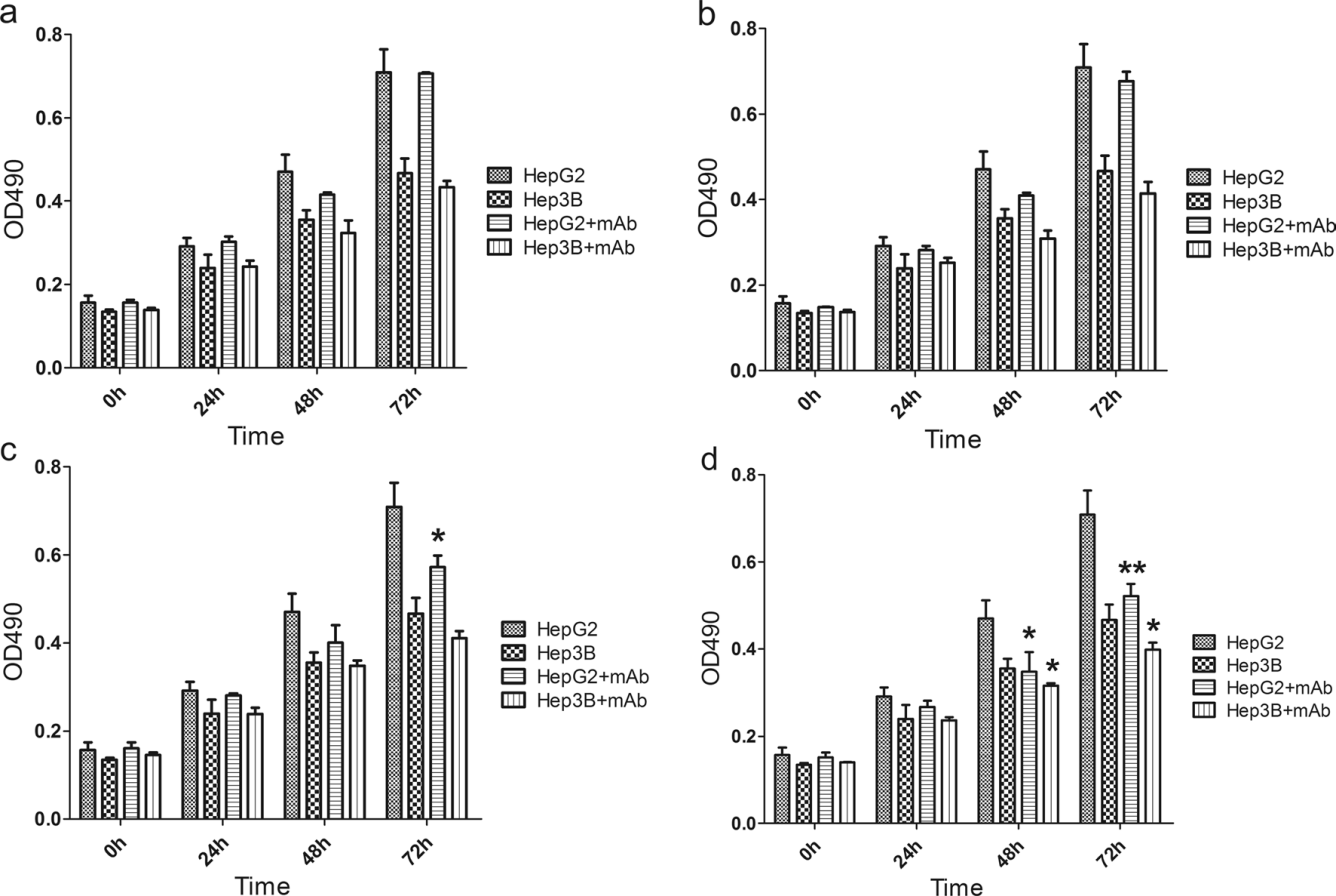
Effect of anti-NEK2 mAb on tumor cell proliferation by MTT assay. Anti-NEK2 mAb at final concentrations of 5 µg/mL, 25 µg/mL, 50 µg/mL and 100 µg/mL was added into hepatocellular carcinoma cells HepG2 and Hep3B for co-culture at 0 h, 24 h, 48 h and 72 h, respectively, and set up HepG2 and Hep3B cells without antibody as control. **a** The final concentration of anti-NEK2 mAb was 5 µg/mL. **b** The final concentration of anti-NEK2 mAb was 25 µg/mL. **c** The final concentration of anti-NEK2 mAb was 50 μg/mL (*P < 0.05). **d** The final concentration of anti-NEK2 mAb was 100 μg/mL (*p < 0.05, **p < 0.01)

The sensitivity of different final antibody concentrations to different HCC cells was different. We found that when HepG2 and Hep3B HCC cells were treated with the anti-NEK2 3A3 mAb at final concentrations of 5 µg/mL and 25 µg/mL, respectively, cell proliferation was not affected (*P* > 0.05) (Fig. 4a, b). The proliferation of HepG2 cells was inhibited after 72 h of culture with the anti-NEK2 3A3 mAb at a final concentration of 50 µg/mL (*P* < 0.05) (Fig. 4c), with an inhibition rate of 19.24%. HepG2 and Hep3B cells cultured for 48 h and 72 h with a final concentration of 100 µg/mL anti-NEK2 3A3 mAb were significantly inhibited (*P* < 0.05) (Fig. 4d). After 48 h, the inhibition rates of the HepG2 and Hep3B cells were 25.99% and 11.06%, respectively. After 72 h, the inhibition rates of the HepG2 and Hep3B cells were 26.34% and 14.43%, respectively. These results showed that the anti-NEK2 3A3 mAb could inhibit the proliferation of HCC cells.

**Fig. 5:**
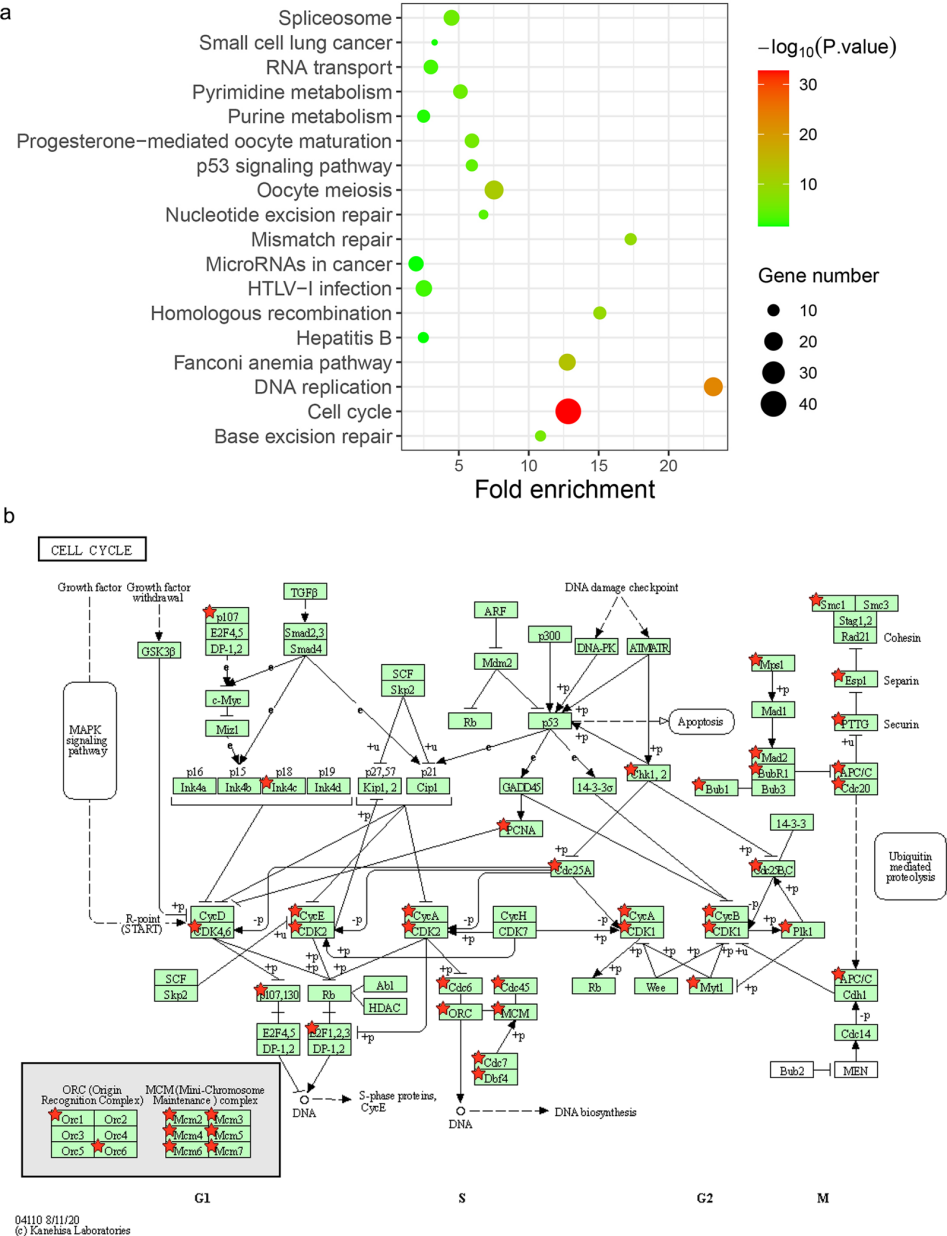
KEGG pathway enrichment analysis and positional relationships of NEK2-associated genes in the cell cycle. **a** Co-expressed genes of NEK2 were analyzed from TCGA using cBioPortal (http://www.cbioportal). **b** KEGG pathways related to the top 500 co-expressed genes of NEK2 were analyzed by DAVID (https://david.ncifcrf.gov/) online software. The NEK2 gene in the cell cycle pathway are brought into the website related to the KEGG pathway to generate a road map

To better explore the potential mechanism of anti-NEK2 mAb on the proliferation of HCC cells, based on the bioinformatics analysis of the cBioPortal database, we found the top 500 genes that were positively related to NEK2 and used the DAVID database for pathway analysis, and the results are shown in Fig. 5. NEK2 might affected pathways of the cell cycle (Fig. 5a). We identified cell cycle regulation pathways that were mainly enriched in co-expressed genes of NEK2, and we found that the co-expressed genes were mainly detected in the G2/M and S phases of the cell cycle, with a few co-expressed genes involved in the regulation of the G1 phase. Thus, NEK2 mainly promoted the transition from S phase to G2/M phase and played an important role in promoting the proliferation of HCC cells (Fig. 5b).

## Discussion

The application of recombinant technology for the expression of exogenous proteins in microorganisms is common in the development and utilization of modern biotechnology. At present, many studies have expressed exogenous genes in prokaryotic expression systems. *E. coli* is often used as a mature bacterium because of its clear genetic background, fast reproduction, high yield, simple operation, mature technology, easy purification, and advantages of easy cultivation, and it has been widely applied in heterologous protein expression of host cells [18]. In this study, the NEK2 protein was expressed through a prokaryotic expression system. To efficiently obtain a large amount of protein, we optimized the protein expression conditions, and the main factors affecting protein expression were temperature, IPTG concentration and induction time [19–21]. We found that the best NEK2 protein yield could be obtained when the recombinant expression vector was induced by 0.2 mmol/L IPTG at 37 °C for 8 h. The NEK2 protein in this study was in inclusion bodies. Inclusion body proteins usually require nickel ion affinity chromatography columns for purification [22]. However, the operation is complicated, time-consuming, and laborious, and the purified protein needs to be dialyzed. Therefore, we adopted a highly efficient, economic and convenient method, KCl staining, which can maintain the antigenicity of the original protein to obtain immunized animals and prepare antibodies.

Anti-NEK2 3A3 mAb was prepared and purified, and the antibody titer was above 1:729,000. The antibody isotype was IgG1, and K_aff_ was 6.0 × 10^8^ L/mol. According to Goding [23], K_aff_ has a high affinity at 10^7^ ~ 10^12^ L/mol. Therefore, the anti-NEK2 3A3 mAb prepared in this study had a high affinity, suggesting that the anti-NEK2 mAb has good clinical application potential. Affinity is one of the important parameters to determine the properties of antibodies. It indicates the degree of affinity binding between antibodies and antigens. Antibodies with a high affinity play an important role in guiding diagnosis and treatment.

In addition, the specificity of monoclonal antibodies is one of their most important properties. In this study, the specificity of the anti-NEK2 3A3 mAb was detected by Western blots, iELISAs, immunofluorescence and immunocytochemical staining. The results showed that the anti-NEK2 3A3 mAb could specifically recognize exogenous and endogenous NEK2 proteins, indicating that the antibody had good antigen specificity. Wang [24] prepared an anti-ω-CTX MVIIA monoclonal antibody with high affinity and specificity and applied it to the development of ELISA kits and immunocolloidal gold test strips. The detection limits were 0.14 μg/mL and 1 μg/mL, respectively, and the linear range of the ELISA was 0.20 ~ 7.22 μg/mL, indicating it can be used as a rapid detection method for screening actual ω-CTX MVIIA samples. In this study, the prepared anti-NEK2 3A3 mAb was applied to detect the subcellular localization of NEK2 protein by immunofluorescence assays, which showed that the NEK2 protein was mainly located in the cytoplasm. We used an anti-NEK2 mAb in an immunocytochemistry staining assay to detect the expression of the NEK2 protein in HL7702, HepG2 and Hep3B cells. The results showed that the expression of NEK2 in the HepG2 and Hep3B HCC cells was significantly higher than that in the normal liver HL7702 cells, and the protein was mainly located in the cytoplasm, which was consistent with the immunohistochemical results of our previous experiment using a mouse anti-human NEK2 monoclonal antibody (Abcam, USA) [9]. The anti-NEK2 3A3 mAb prepared in this study can be used in immunofluorescence and immunocytochemical staining assays, which provides an experimental basis for the establishment of a NEK2 rapid detection kit and immunocolloidal gold detection strip.

Monoclonal antibodies can be used not only for the diagnosis of diseases but also for the treatment of tumors. At present, a variety of monoclonal antibodies have been used in the clinical treatment of various cancers. Monoclonal antibodies targeting the epidermal growth factor receptor (EGFR), including cetuximab and panitumumab, were effective against metastatic colorectal cancer, and GC1118 (a novel, fully humanized anti-EGFR IgG1 antibody) alone had a better antitumor effect [25]. One study showed that specific antibodies had antitumor effects [26]. To explore the effect of the anti-NEK2 mAb specifically binding corresponding antigens on the growth and proliferation of HCC cells, we used HepG2 and Hep3B cells with high expression of NEK2 protein in this study, and the prepared anti-NEK2 3A3 mAb was co-cultured with HCC cells to observe the effect of antibodies on HCC cells. As determined by the MTT method, an anti-NEK2 mAb inhibited the proliferation of HCC cells in a dose-dependent manner. We hypothesize that the anti-NEK2 mAb may enter cells through endocytosis and bind specifically to NEK2 in cells, thereby inhibiting cell proliferation [27]. This hypothesis suggests that anti-NEK2 mAb has a potential tumor suppressive effect on liver cancer with high expression of NEK2 protein. Targeted inhibition of the NEK2 protein can effectively inhibit the growth of liver cancer cells. To explore the mechanism by which the anti-NEK2 mAb affected the occurrence and development of HCC cell proliferation, we used bioinformatics correlation analysis to identify the top 500 genes that were positively related to NEK2 [28]. KEGG pathway analysis showed that the expression of NEK2 might affects pathways related to the cell cycle. Some studies have shown that the NEK family is mainly involved in the regulation of the G2 to M checkpoint in mitosis. NEK2 is regulated by the cell cycle, and its expression and activity in the S and G2 phases peaked [29]. Our results showed that NEK2-related genes were mainly enriched in the S and G2 phases and might act as tumor-promoting genes by regulating the S/G2 phase transition of HCC cells. The occurrence and development of tumors are closely related to the abnormal expression of cell cycle genes. Therefore, anti-NEK2 mAb may be a potential effective method of targeted therapy for liver cancer.

We produced the recombinant fusion protein NEK2 in *E. coli* and prepared a monoclonal antibody using the NEK2 protein for the first time. Moreover, an anti-NEK2 mAb with high potency, high affinity and high specificity was prepared in this study, providing an experimental basis for the establishment of a NEK2 rapid diagnostic kit and immunocolloidal gold detection strip. These results indicate that the anti-NEK2 3A3 mAb can inhibit the proliferation of HCC cells, which provides a basis for research on and development of antitumor drugs.

## Conclusions

In this study, NEK2 protein was cloned and expressed with an effective and reliable method and injected into BALB/c mice to prepare an anti-NEK2 mAb. An anti-NEK2 mAb with high potency, high affinity and high specificity was obtained and proved suitable for both immunofluorescence and immunocytochemical staining assays. Considering that NEK2 may be a new target for cancer treatment, our research provides an alternative method for the rapid preparation of anti-NEK2 mAb for clinical applications.

## Supplementary Information

**Additional file 1.** Sequence alignment results. N22: Sequence of the positive clonal bacterial solution. NEK2: Sequence of NEK2 gene (NCBI Gene ID: 4751).

**Additional file 2.** **Fig. S1.** The full length (uncut) gel image of Fig. 1a. PCR amplification and colony PCR product of the NEK2 gene. **Fig. S2.** The full length (uncut) gel image of Fig. 1b. BamHI and Sa1I enzymatic digestion of the recombinant plasmid. **Fig. S3.** The full length (uncut) gel image of Fig. 1c. Expression of the recombinant human NEK2 protein. **Fig. S4.** The full length (uncut) gel image of Fig. 1d. Expression of recombinant human NEK2 protein at different temperatures. **Fig. S5.** The full length (uncut) gel image of Fig. 1e. Expression of recombinant human NEK2 protein at different IPTG concentrations. **Fig. S6.** The full length (uncut) gel image of Fig. 1f. Expression of recombinant human NEK2 protein at different times. **Fig. S7.** The full length (uncut) gel image of Fig. 1g. Recombinant human NEK2 protein purification. **Fig. S8.** The full length (uncut) blot image of Fig. 1h. Detection ofthe expression of recombinant protein by Western blot. **Fig. S9.** The full length (uncut) gel image of Fig. 2b. Anti-NEK2 mAb purification. **Fig. S10.** The full length (uncut) blot image of Fig. 3a. Analysis of the antigenic specificity of anti-NEK2 mAb by Western blot.

## Data Availability

All authors declare that the data supporting the findings of this study are available within the article and supplementary file.
