# Supplementary material for: Preparation of an anti-NEK2 monoclonal antibody and its application in liver cancer
Source: BMC Biotechnol. 2021 Oct 27;21:62. doi: 10.1186/s12896-021-00717-3 (PMC8549277; doi:10.1186/s12896-021-00717-3)
Supplement: Supplementary file 1 — Additional file 1. Sequence alignment results. N22: Sequence of the positive clonal bacterial solution. NEK2: Sequence of NEK2 gene (NCBI Gene ID: 4751). [file 12896_2021_717_MOESM1_ESM.pdf]

Preparation of an anti-NEK2 monoclonal antibody and its application in liver cancer

Qiuli Chen[1#], Hui Li[1#], Lichao Yang[1], Sha Wen[2], Xuejing Huang[2], Jiajuan Liu[2], Xiaoping Guo[2], Bing Hu[2], Gang Li[1*], Min He[1,2,3*]

1 School of Public Health, Guangxi Medical University, Nanning, 530021, China

2 Laboratory Animal Center of Guangxi Medical University, Nanning, 530021, China

3 Key Laboratory of High-Incidence-Tumor Prevention & Treatment (Guangxi Medical University), Ministry of Education, Nanning, 530021, China

[#]Contributed equally

*Corresponding authors: Min He hemin@gxmu.edu.cn

Gang Li ligang@gxmu.edu.cn

Data S1 Sequence alignment results

N22: Sequence of the positive clonal bacterial solution. NEK2: Sequence of NEK2 gene (NCBI Gene ID: 4751).

```
                 1                                                50
    N22       (1) CCCTCTAGAAATAATTTTGTTTAACTTTAAGAAGGAGATATACATATGCA
   NEK2       (1) --------------------------------------------------
Consensus     (1)
                 51                                               100
    N22      (51) CCATCATCATCATCATTCTTCTGGTCTGGTGCCACGCGGTTCTGGTATGA
   NEK2       (1) --------------------------------------------------
Consensus    (51)
                 101                                              150
    N22     (101) AAGAAACCGCTGCTGCTAAATTCGAACGCCAGCACATGGACAGCCCAGAT
   NEK2       (1) --------------------------------------------------
Consensus   (101)
                 151                                              200
    N22     (151) CTGGGTACCGACGACGACGACAAGGCCATGGCTGATATCGGATCCATGCC
   NEK2       (1) ---------------------------------------------ATGCC
Consensus   (151)                                              ATGCC
                 201                                              250
    N22     (201) TTCCCGGGCTGAGGACTATGAAGTGTTGTACACCATTGGCACAGGCTCCT
   NEK2       (6) TTCCCGGGCTGAGGACTATGAAGTGTTGTACACCATTGGCACAGGCTCCT
Consensus   (201) TTCCCGGGCTGAGGACTATGAAGTGTTGTACACCATTGGCACAGGCTCCT
                 251                                              300
    N22     (251) ACGGCCGCTGCCAGAAGATCCGGAGGAAGAGTGATGGCAAGATATTAGTT
   NEK2      (56) ACGGCCGCTGCCAGAAGATCCGGAGGAAGAGTGATGGCAAGATATTAGTT
Consensus   (251) ACGGCCGCTGCCAGAAGATCCGGAGGAAGAGTGATGGCAAGATATTAGTT
                 301                                              350
    N22     (301) TGGAAAGAACTTGACTATGGCTCCATGACAGAAGCTGAGAAACAGATGCT
   NEK2     (106) TGGAAAGAACTTGACTATGGCTCCATGACAGAAGCTGAGAAACAGATGCT
Consensus   (301) TGGAAAGAACTTGACTATGGCTCCATGACAGAAGCTGAGAAACAGATGCT
                 351                                              400
    N22     (351) TGTTTCTGAAGTGAATTTGCTTCGTGAACTGAAACATCCAAACATCGTTC
   NEK2     (156) TGTTTCTGAAGTGAATTTGCTTCGTGAACTGAAACATCCAAACATCGTTC
Consensus   (351) TGTTTCTGAAGTGAATTTGCTTCGTGAACTGAAACATCCAAACATCGTTC
                 401                                              450
    N22     (401) GTTACTATGATCGGATTATTGACCGGACCAATACAACACTGTACATTGTA
   NEK2     (206) GTTACTATGATCGGATTATTGACCGGACCAATACAACACTGTACATTGTA
Consensus   (401) GTTACTATGATCGGATTATTGACCGGACCAATACAACACTGTACATTGTA
                 451                                              500
```

```
N22        (451) ATGGAATATTGTGAAGGAGGGGATCTGGCTAGTGTAATTACAAAGGGAAC
NEK2       (256) ATGGAATATTGTGAAGGAGGGGATCTGGCTAGTGTAATTACAAAGGGAAC
Consensus  (451) ATGGAATATTGTGAAGGAGGGGATCTGGCTAGTGTAATTACAAAGGGAAC
                 501                                              550
N22        (501) CAAGGAAAGGCAATACTTAGATGAAGAGTTTGTTCTTCGAGTGATGACTC
NEK2       (306) CAAGGAAAGGCAATACTTAGATGAAGAGTTTGTTCTTCGAGTGATGACTC
Consensus  (501) CAAGGAAAGGCAATACTTAGATGAAGAGTTTGTTCTTCGAGTGATGACTC
                 551                                              600
N22        (551) AGTTGACTCTGGCCCTGAAGGAATGCCACAGACGAAGTGATGGTGGTCAT
NEK2       (356) AGTTGACTCTGGCCCTGAAGGAATGCCACAGACGAAGTGATGGTGGTCAT
Consensus  (551) AGTTGACTCTGGCCCTGAAGGAATGCCACAGACGAAGTGATGGTGGTCAT
                 601                                              650
N22        (601) ACCGTATTGCATCGGGATCTGAAACCAGCCAATGTTTTCCTGGATGGCAA
NEK2       (406) ACCGTATTGCATCGGGATCTGAAACCAGCCAATGTTTTCCTGGATGGCAA
Consensus  (601) ACCGTATTGCATCGGGATCTGAAACCAGCCAATGTTTTCCTGGATGGCAA
                 651                                              700
N22        (651) GCAAAACGTCAAGCTTGGAGACTTTGGGCTAGCTAGAATATTAAACCATG
NEK2       (456) GCAAAACGTCAAGCTTGGAGACTTTGGGCTAGCTAGAATATTAAACCATG
Consensus  (651) GCAAAACGTCAAGCTTGGAGACTTTGGGCTAGCTAGAATATTAAACCATG
                 701                                              750
N22        (701) ACACGAGTTTTGCAAAAACATTTGTTGGCACACCTTATTACATGTCTCCT
NEK2       (506) ACACGAGTTTTGCAAAAACATTTGTTGGCACACCTTATTACATGTCTCCT
Consensus  (701) ACACGAGTTTTGCAAAAACATTTGTTGGCACACCTTATTACATGTCTCCT
                 751                                              800
N22        (751) GAACAAATGAATCGCATGTCCTACAATGAGAAATCAGATATCTGGTCATT
NEK2       (556) GAACAAATGAATCGCATGTCCTACAATGAGAAATCAGATATCTGGTCATT
Consensus  (751) GAACAAATGAATCGCATGTCCTACAATGAGAAATCAGATATCTGGTCATT
                 801                                              850
N22        (801) GGGCTGCTTGCTGTATGAGTTATGTGCATTAATGCCTCCATTTACAGCTT
NEK2       (606) GGGCTGCTTGCTGTATGAGTTATGTGCATTAATGCCTCCATTTACAGCTT
Consensus  (801) GGGCTGCTTGCTGTATGAGTTATGTGCATTAATGCCTCCATTTACAGCTT
                 851                                              900
N22        (851) TTAGCCAGAAAGAACTCGCTGGGAAAATCAGAGAAGGCAAATTCAGGCGA
NEK2       (656) TTAGCCAGAAAGAACTCGCTGGGAAAATCAGAGAAGGCAAATTCAGGCGA
Consensus  (851) TTAGCCAGAAAGAACTCGCTGGGAAAATCAGAGAAGGCAAATTCAGGCGA
                 901                                              950
N22        (901) ATTCCATACCGTTACTCTGATGAATTGAATGAAATTATTACGAGGATGTT
NEK2       (706) ATTCCATACCGTTACTCTGATGAATTGAATGAAATTATTACGAGGATGTT
Consensus  (901) ATTCCATACCGTTACTCTGATGAATTGAATGAAATTATTACGAGGATGTT
                 951                                             1000
N22        (951) AAACTTAAAGGATTACCATCGACCTTCTGTTGAAGAAATTCTTGAGAACC
```

```
     NEK2   (756) AAACTTAAAGGATTACCATCGACCTTCTGTTGAAGAAATTCTTGAGAACC
Consensus   (951) AAACTTAAAGGATTACCATCGACCTTCTGTTGAAGAAATTCTTGAGAACC
                  1001                                              1050
      N22  (1001) CTTTAATAGCAGATTTGGTTGCAGACGAGCAAAGAAGAAATCTTGAGAGA
     NEK2   (806) CTTTAATAGCAGATTTGGTTGCAGACGAGCAAAGAAGAAATCTTGAGAGA
Consensus  (1001) CTTTAATAGCAGATTTGGTTGCAGACGAGCAAAGAAGAAATCTTGAGAGA
                  1051                                              1100
      N22  (1051) AGAGGGCGACAATTAGGAGAGCCAGAAAAATCGCAGGATTCCAGCCCTGT
     NEK2   (856) AGAGGGCGACAATTAGGAGAGCCAGAAAAATCGCAGGATTCCAGCCCTGT
Consensus  (1051) AGAGGGCGACAATTAGGAGAGCCAGAAAAATCGCAGGATTCCAGCCCTGT
                  1101                                              1150
      N22  (1101) ATTGAGTGAGCTGAAACTGAAGGAAATTCAGTTACAGGAGCGAGAGCGAG
     NEK2   (906) ATTGAGTGAGCTGAAACTGAAGGAAATTCAGTTACAGGAGCGAGAGCGAG
Consensus  (1101) ATTGAGTGAGCTGAAACTGAAGGAAATTCAGTTACAGGAGCGAGAGCGAG
                  1151                                              1200
      N22  (1151) CTCTCAAAGCAAGAGAAGAAAGATTGGAGCAGAAAGAACAGGAGCTTTGT
     NEK2   (956) CTCTCAAAGCAAGAGAAGAAAGATTGGAGCAGAAAGAACAGGAGCTTTGT
Consensus  (1151) CTCTCAAAGCAAGAGAAGAAAGATTGGAGCAGAAAGAACAGGAGCTTTGT
                  1201                                              1250
      N22  (1201) GTTCGTGAGAGACTAGCAGAGGACAAACTGGCTAGAGCAGAAAATCTGTT
     NEK2  (1006) GTTCGTGAGAGACTAGCAGAGGACAAACTGGCTAGAGCAGAAAATCTGTT
Consensus  (1201) GTTCGTGAGAGACTAGCAGAGGACAAACTGGCTAGAGCAGAAAATCTGTT
                  1251                                              1300
      N22  (1251) GAAGAACTACAGCTTGCTAAAGGAACGGAAGTTCCTGTCTCTGGCAAGTA
     NEK2  (1056) GAAGAACTACAGCTTGCTAAAGGAACGGAAGTTCCTGTCTCTGGCAAGTA
Consensus  (1251) GAAGAACTACAGCTTGCTAAAGGAACGGAAGTTCCTGTCTCTGGCAAGTA
                  1301                                              1350
      N22  (1301) ATCCAGAACTTCTTAATCTTCCATCCTCAGTAATTAAGAAGAAAGTTCAT
     NEK2  (1106) ATCCAGAACTTCTTAATCTTCCATCCTCAGTAATTAAGAAGAAAGTTCAT
Consensus  (1301) ATCCAGAACTTCTTAATCTTCCATCCTCAGTAATTAAGAAGAAAGTTCAT
                  1351                                              1400
      N22  (1351) TTCAGTGGGGAAAGTAAAGAGAACATCATGAGGAGTGAGAATTCTGAGAG
     NEK2  (1156) TTCAGTGGGGAAAGTAAAGAGAACATCATGAGGAGTGAGAATTCTGAGAG
Consensus  (1351) TTCAGTGGGGAAAGTAAAGAGAACATCATGAGGAGTGAGAATTCTGAGAG
                  1401                                              1450
      N22  (1401) TCAGCTCACATCTAAGTCCAAGTGCAAGGACCTGAAGAAAAGGCTTCACG
     NEK2  (1206) TCAGCTCACATCTAAGTCCAAGTGCAAGGACCTGAAGAAAAGGCTTCACG
Consensus  (1401) TCAGCTCACATCTAAGTCCAAGTGCAAGGACCTGAAGAAAAGGCTTCACG
                  1451                                              1500
      N22  (1451) CTGCCCAGCTGCGGGCTCAAGCCCTGTCAGATATTGAGAAAAATTACCAA
     NEK2  (1256) CTGCCCAGCTGCGGGCTCAAGCCCTGTCAGATATTGAGAAAAATTACCAA
```

```
Consensus  (1451) CTGCCCAGCTGCGGGCTCAAGCCCTGTCAGATATTGAGAAAAATTACCAA
                  1501                                              1550
      N22  (1501) CTGAAAAGCAGACAGATCCTGGGCATGCGCTAGGTCGACAAGCTTGCGGC
     NEK2  (1306) CTGAAAAGCAGACAGATCCTGGGCATGCGCTAG-----------------
Consensus  (1501) CTGAAAAGCAGACAGATCCTGGGCATGCGCTAG
                  1551                                              1600
      N22  (1551) CGCACTCGAGCACCACCACCACCACCACTGAGATCCGGCTGCTAACAAAG
     NEK2  (1339) --------------------------------------------------
Consensus  (1551)
                  1601                                              1650
      N22  (1601) CCCGAAAGGAAGCTGAGTTGGCTGCTGCCACCGCTGAGCAATAACTAGCA
     NEK2  (1339) --------------------------------------------------
Consensus  (1601)
                  1651                                              1700
      N22  (1651) TAACCCCTTGGGGCCTCTAAACGGGTCTTGAGGGGTTTTTTGCTGAAAGG
     NEK2  (1339) --------------------------------------------------
Consensus  (1651)
                  1701                                              1750
      N22  (1701) AGGAACTATATCCGGATTGGCGAATGGGACGCGCCCTGTAGCGGCGCATT
     NEK2  (1339) --------------------------------------------------
Consensus  (1701)
                  1751                                              1800
      N22  (1751) AAGCGCGGGGGGTGTGGGGTTACGCGCAGCGTGACCGCTACCTTGCCGCG
     NEK2  (1339) --------------------------------------------------
Consensus  (1751)
                  1801             1819
      N22  (1801) CCTAGCGCCGCTCTTTCGT
     NEK2  (1339) -------------------
Consensus  (1801)
```
