# Supplementary material for: Preparation of an anti-NEK2 monoclonal antibody and its application in liver cancer
Source: BMC Biotechnol. 2021 Oct 27;21:62. doi: 10.1186/s12896-021-00717-3 (PMC8549277; doi:10.1186/s12896-021-00717-3)
Supplement: Supplementary file 2 — Additional file 2. Fig. S1. The full length (uncut) gel image of Fig. 1a. PCR amplification and colony PCR product of the NEK2 gene. Fig. S2. The full length (uncut) gel image of Fig. 1b. BamHI and Sa1I enzymatic digestion of the recombinant plasmid. Fig. S3. The full length (uncut) gel image of Fig. 1c. Expression of the recombinant human NEK2 protein. Fig. S4. The full length (uncut) gel image of Fig. 1d. Expression of recombinant human NEK2 protein at different temperatures. Fig. S5. The full length (uncut) gel image of Fig. 1e. Expression of recombinant human NEK2 protein at different IPTG concentrations. Fig. S6. The full length (uncut) gel image of Fig. 1f. Expression of recombinant human NEK2 protein at different times. Fig. S7. The full length (uncut) gel image of Fig. 1g. Recombinant human NEK2 protein purification. Fig. S8. The full length (uncut) blot image of Fig. 1h. Detection ofthe expression of recombinant protein by Western blot. Fig. S9. The full length (uncut) gel image of Fig. 2b. Anti-NEK2 mAb purification. Fig. S10. The full length (uncut) blot image of Fig. 3a. Analysis of the antigenic specificity of anti-NEK2 mAb by Western blot. [file 12896_2021_717_MOESM2_ESM.pdf]

Preparation of an anti-NEK2 monoclonal antibody and its application in liver cancer

Qiuli Chen[1 #], Hui Li[1 #], Lichao Yang[1], Sha Wen[2], Xuejing Huang[2], Jiajuan Liu[2], Xiaoping Guo[2], Bing Hu[2], Gang Li[1*], Min He[1,2,3*]

1 School of Public Health, Guangxi Medical University, Nanning, 530021, China

2 Laboratory Animal Center of Guangxi Medical University, Nanning, 530021, China

3 Key Laboratory of High-Incidence-Tumor Prevention & Treatment (Guangxi Medical University), Ministry of Education, Nanning, 530021, China

[#]Contributed equally

*Corresponding authors: Min He hemin@gxmu.edu.cn

Gang Li ligang@gxmu.edu.cn

Supplementary figure captions

Fig. S1 The full length (uncut) gel image of Fig. 1a. PCR amplification and colony PCR product of the *NEK2* gene.

Fig. S2 The full length (uncut) gel image of Fig. 1b. BamHI and Sa1I enzymatic digestion of the recombinant plasmid.

Fig. S3 The full length (uncut) gel image of Fig. 1c. Expression of the recombinant human NEK2 protein.

Fig. S4 The full length (uncut) gel image of Fig. 1d. Expression of recombinant human NEK2 protein at different temperatures.

Fig. S5 The full length (uncut) gel image of Fig. 1e. Expression of recombinant human NEK2 protein at different IPTG concentrations.

Fig. S6 The full length (uncut) gel image of Fig. 1f. Expression of recombinant human NEK2 protein at different times.

Fig. S7 The full length (uncut) gel image of Fig. 1g. Recombinant human NEK2 protein purification.

Fig. S8 The full length (uncut) blot image of Fig. 1h. Detection of the expression of recombinant protein by Western blot.

Fig. S9 The full length (uncut) gel image of Fig. 2b. Anti-NEK2 mAb purification.

Fig. S10 The full length (uncut) blot image of Fig. 3a. Analysis of the antigenic specificity of anti-NEK2 mAb by Western blot.

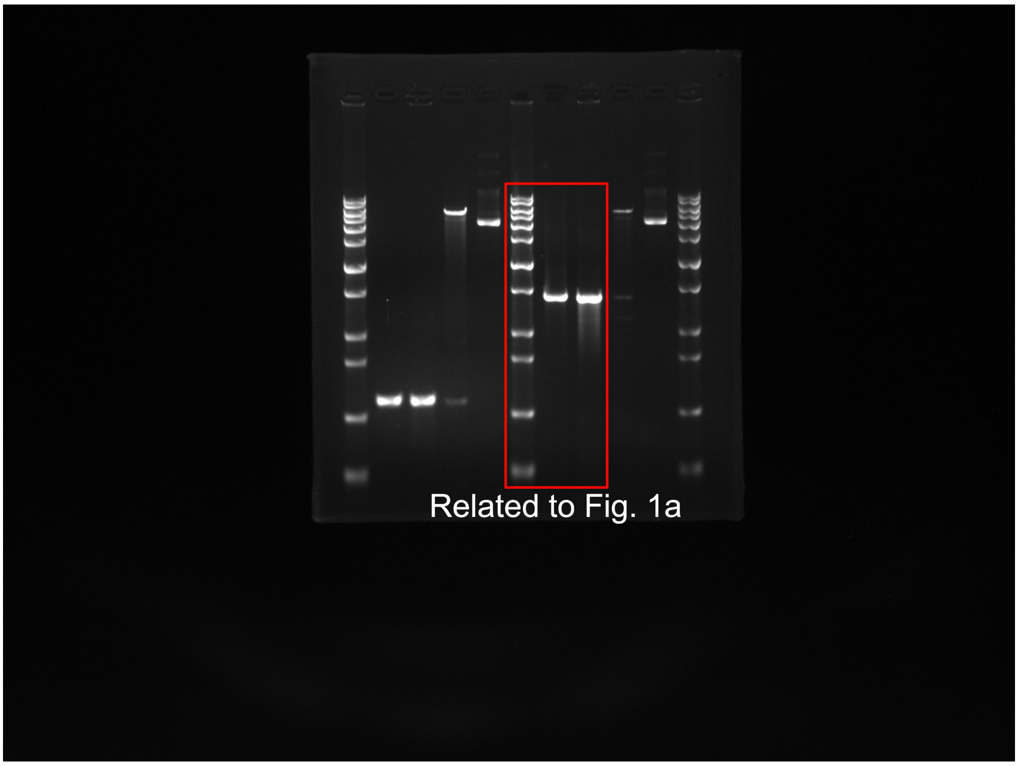

Fig. S1

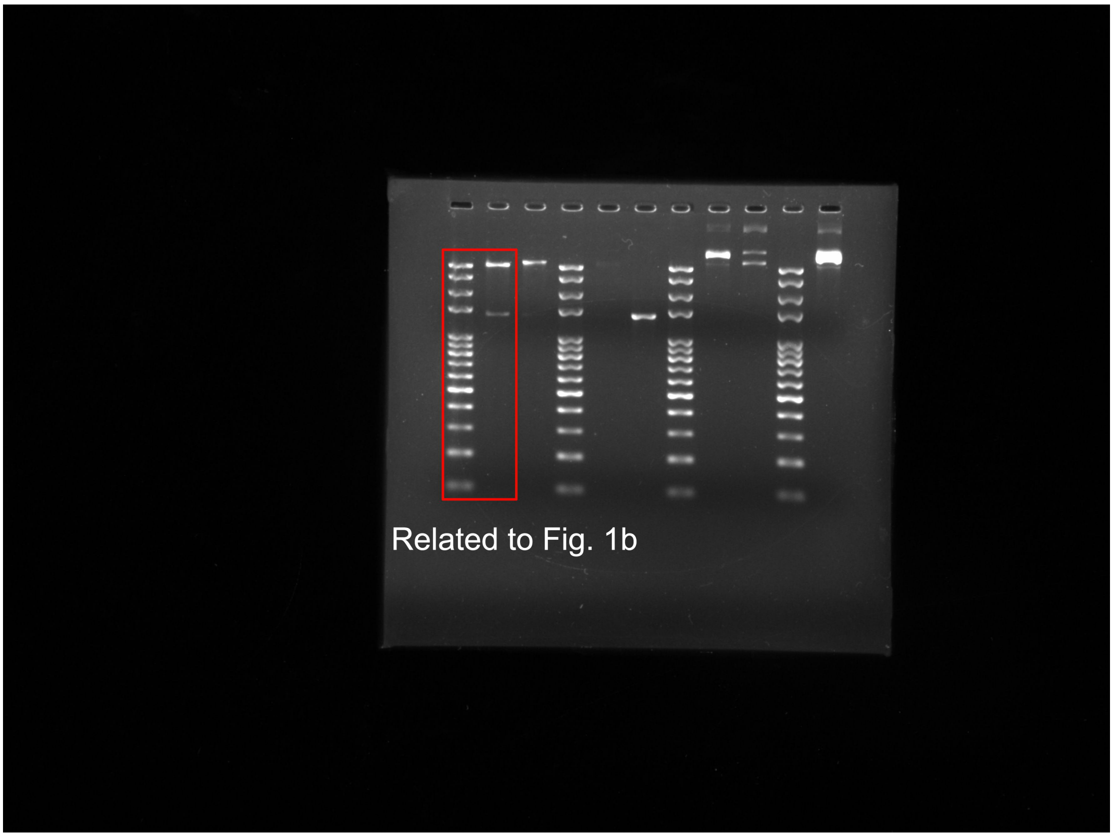

Fig. S2

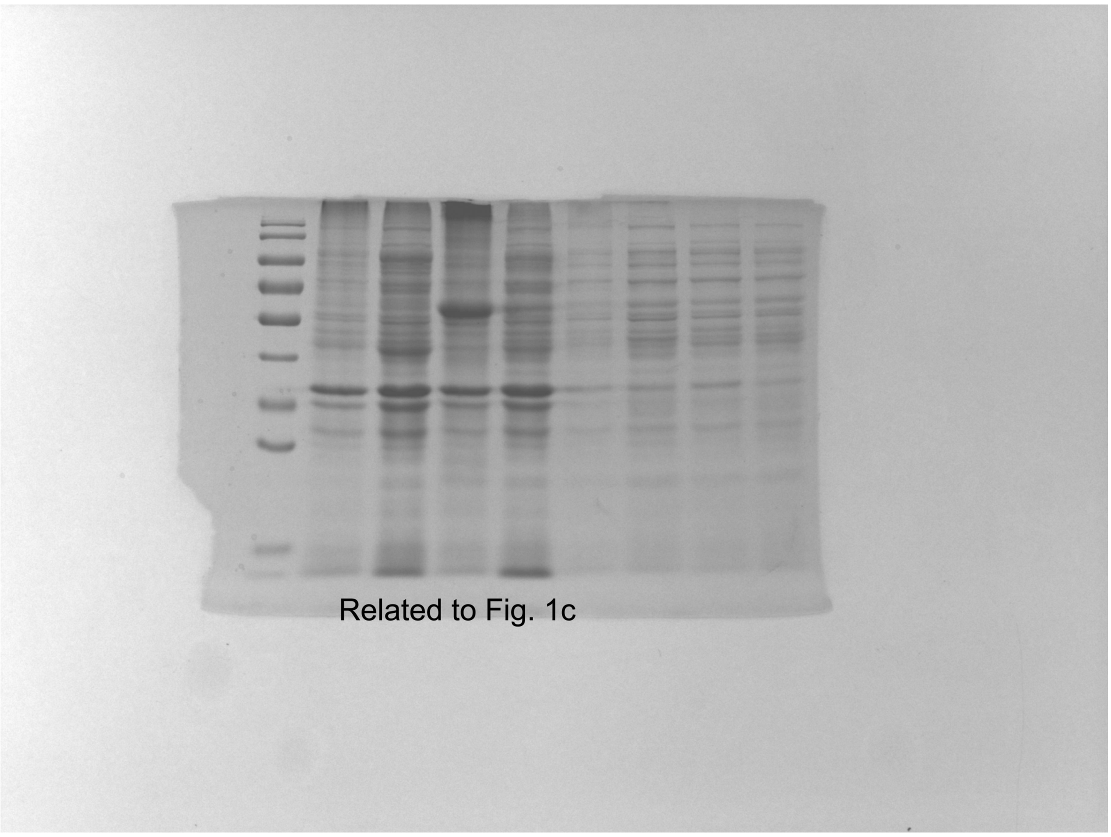

Fig. S3

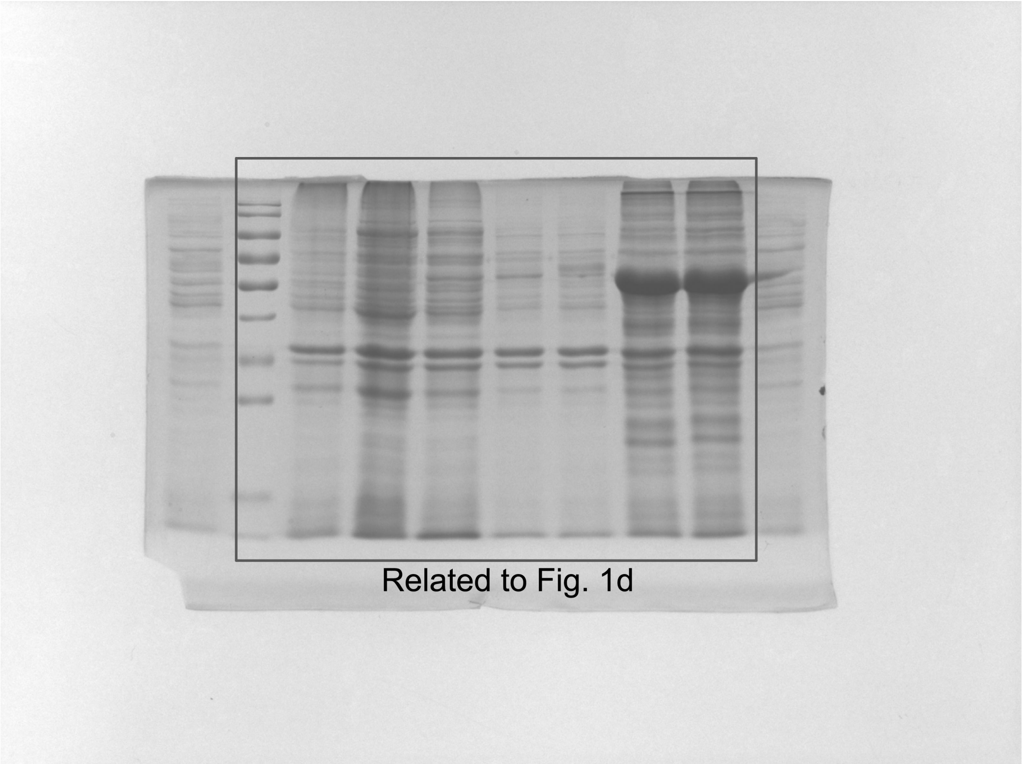

Fig. S4

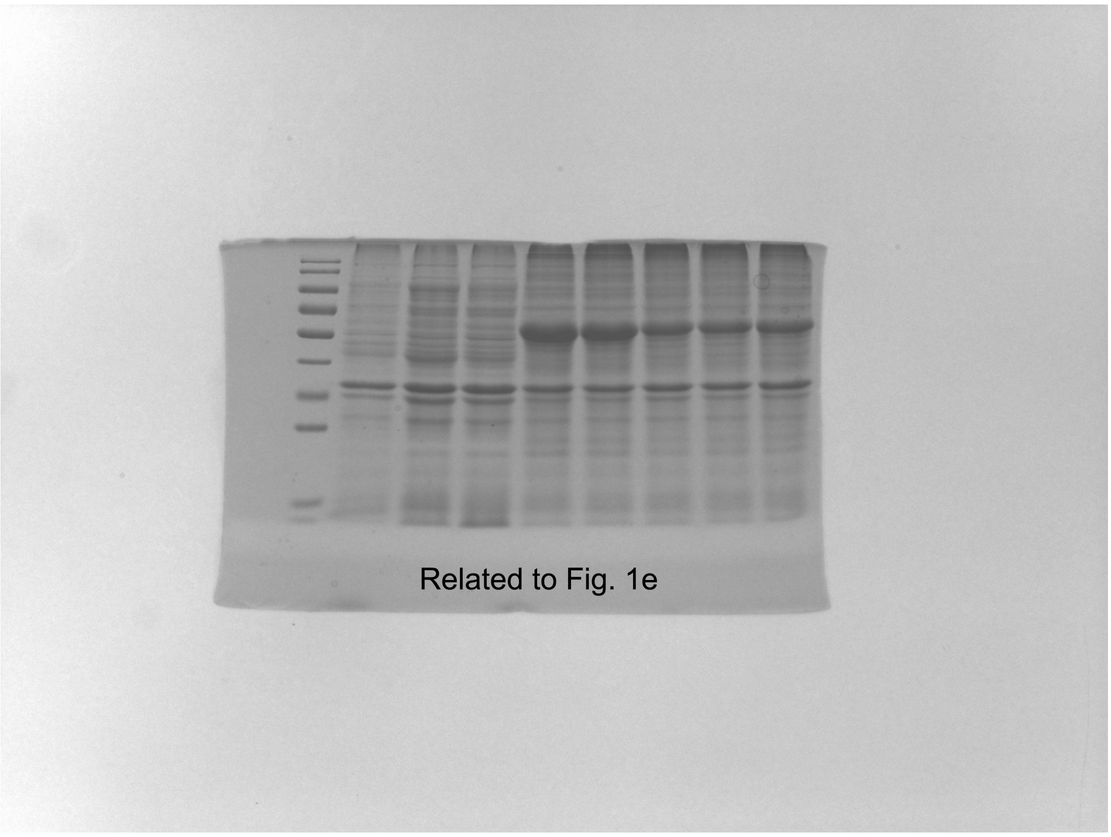

Fig. S5

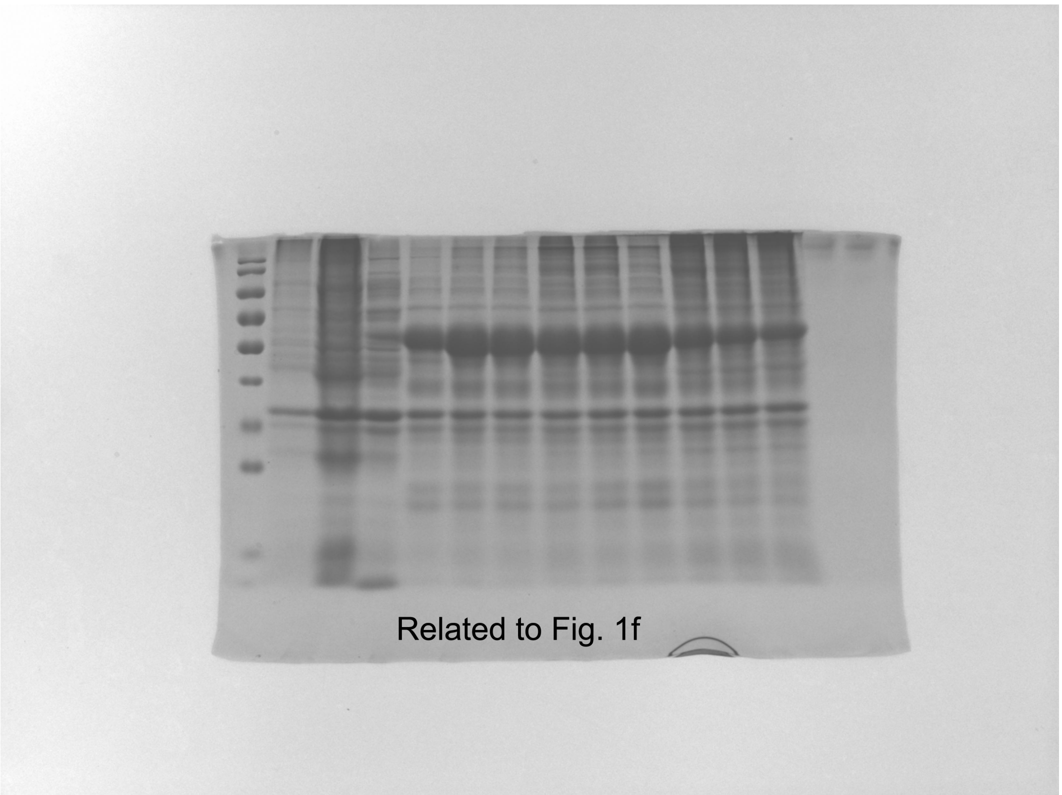

Fig. S6

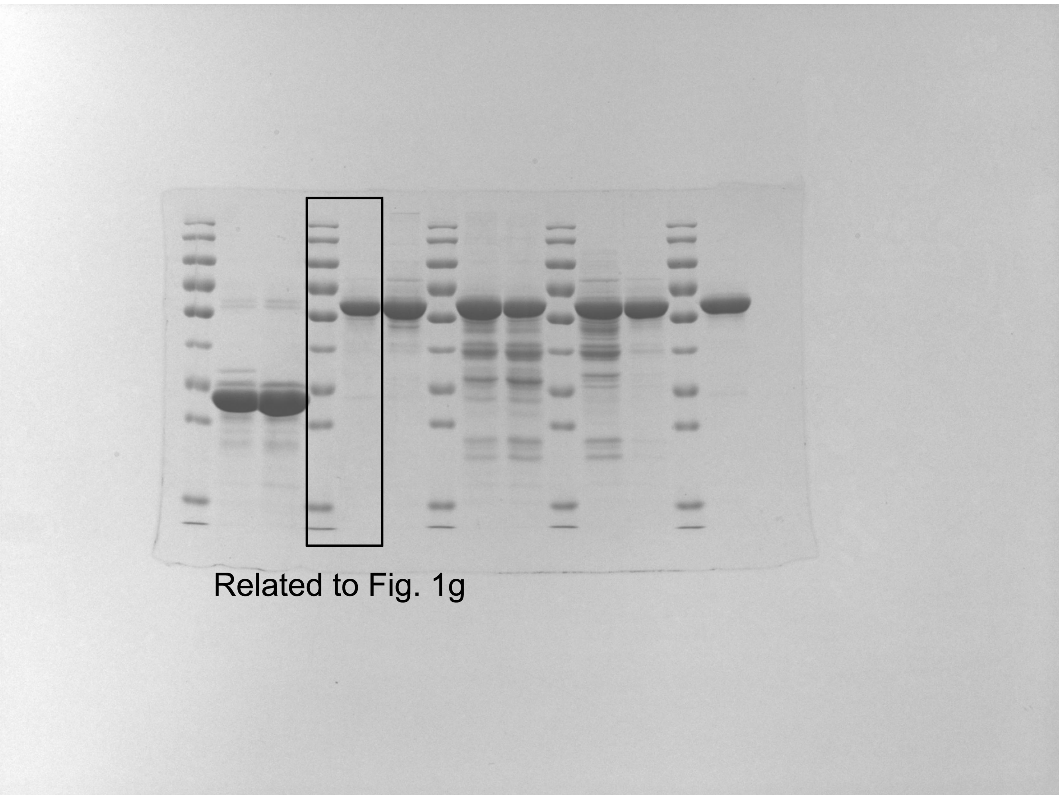

Fig. S7

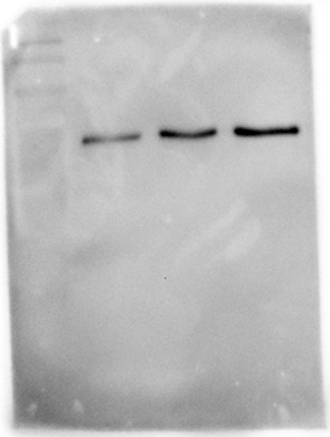

Related to Fig. 1h

Fig. S8

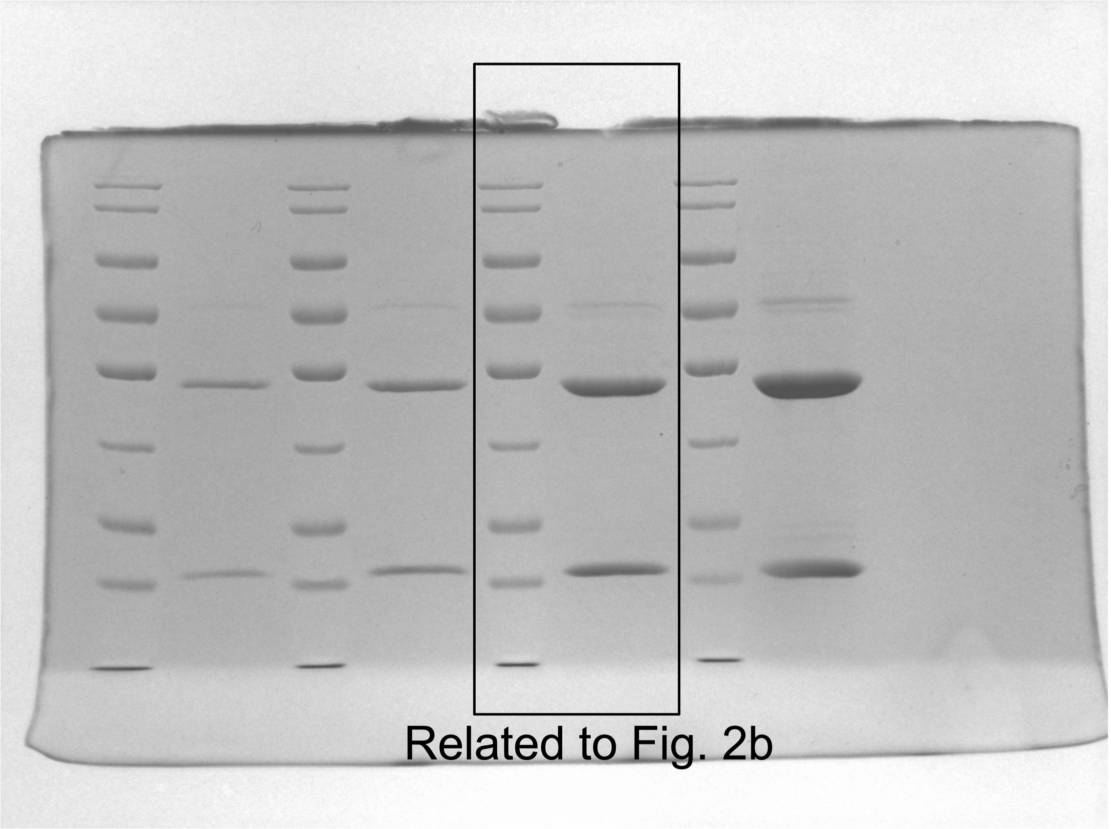

Fig. S9

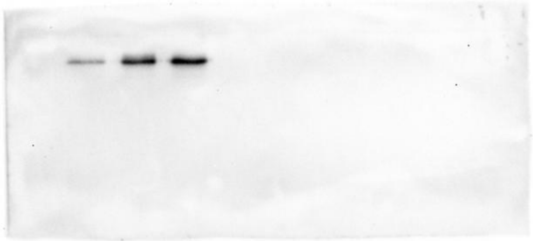

Related to Fig. 3a

Fig. S10
